# Association between hospital volume and in‐hospital mortality following radiofrequency ablation for hepatocellular carcinoma

**DOI:** 10.1002/bjs5.9

**Published:** 2017-07-26

**Authors:** M. Sato, R. Tateishi, H. Yasunaga, H. Matsui, K. Fushimi, H. Ikeda, Y. Yatomi, K. Koike

**Affiliations:** ^1^ Department of Clinical Laboratory Medicine University of Tokyo Tokyo Japan; ^2^ Department of Gastroenterology, Graduate School of Medicine University of Tokyo Tokyo Japan; ^3^ Department of Clinical Epidemiology and Health Economics, School of Public Health University of Tokyo Tokyo Japan; ^4^ Department of Health Policy and Informatics Tokyo Medical and Dental University Graduate School of Medicine Tokyo Japan

## Abstract

**Background:**

Radiofrequency ablation (RFA) is a minimally invasive treatment for hepatocellular carcinoma (HCC). There is increasing evidence of an association between increasing hospital volume and lower postoperative mortality for many surgical procedures, but this is difficult to establish with minimally invasive treatments, where postoperative mortality is low. The aim of this study was to investigate the relationship between hospital volume and in‐hospital mortality following RFA using a Japanese nationwide database.

**Methods:**

Data from the Diagnostic Procedure Combination database were analysed from 1 July 2010 to 31 March 2012. Multivariable logistic regression was used to analyse the relationship between hospital volume and in‐hospital mortality following RFA, with adjustment for patient background.

**Results:**

Some 36 675 patients with HCC were identified in the database. The overall in‐hospital mortality rate from RFA was 0·31 per cent. In‐hospital mortality was significantly higher in low‐volume than high‐volume hospitals (odds ratio 2·57, 95 per cent c.i. 1·61 to 4·09; P < 0·001). Higher in‐hospital mortality was significantly associated with older age and a higher Charlson Co‐morbidity Index score.

**Conclusion:**

RFA for HCC was associated with acceptably low mortality in Japan, but in‐hospital mortality following RFA was affected by hospital procedural volume.

## Introduction

Hepatocellular carcinoma (HCC) is the fifth most frequently diagnosed cancer and the third most common cause of cancer‐related death worldwide^1^. Only 20 per cent of patients with HCC are candidates for resection^2^. Image‐guided minimally invasive techniques have been used widely for the treatment of HCC in the past two decades and radiofrequency ablation (RFA) has yielded promising clinical results, with survival rates comparable to those of hepatectomy^3^. During the past decade, there has been growing interest in the use of RFA in patients with liver tumours considered to be unresectable because of impaired hepatic function or associated co‐morbidities^4^, ^5^, ^6^, ^7^. Surveillance programmes have reduced the proportion of patients presenting with advanced disease and this has also led to an increase in the use of RFA^2^ ^8^.

There has been increasing evidence of an association between hospital volume and postoperative mortality and morbidity following surgical procedures^9^, ^10^, ^11^, although it is difficult to demonstrate such a relationship following minimally invasive treatments such as RFA, largely as a result of very low in‐hospital mortality rates^3^ ^12^.

The aim of this study was to investigate the relationship between hospital volume and in‐hospital mortality following RFA for HCC in a large patient sample identified from a Japanese national inpatient database.

## Methods

The Diagnosis Procedure Combination (DPC) database is a discharge abstract and administrative claims database of inpatient admissions to secondary‐ and tertiary‐care hospitals in Japan^13^, ^14^, ^15^. The database includes data on approximately 7 million inpatients annually, representing approximately 50 per cent of inpatient admissions to such hospitals. The database contains the following information: patient demographics; diagnoses, co‐morbidities at admission and complications after admission (recorded with Japanese text and ICD‐10 codes); therapeutic procedures encoded by original Japanese codes; duration of hospital stay; discharge status including in‐hospital death; and total costs. All 82 academic hospitals in Japan are required to participate in the database, but participation is optional for community hospitals.

The requirement for informed consent was waived for this study because of the anonymous nature of the data. Study approval was obtained from the institutional review board of the University of Tokyo.

Inpatient data for a total of 21 months (1 July 2010 to 31 March 2012) were obtained. Among 12 million inpatients included in the database, patients with a diagnosis of HCC (ICD‐10 code C220) were identified, including those who had undergone RFA. Patients who had undergone transcatheter arterial embolization ahead of RFA were excluded to avoid any influence of embolization‐related liver damage^16^ on in‐hospital mortality following RFA. The Charlson Co‐morbidity Index (CCI), a validated, weighted co‐morbidity score derived from unselected hospital admissions that predicts 1‐year mortality after hospital discharge^17^, was used to adjust for multiple co‐morbidities^18^. Hospital volume was defined as the annual number of RFA procedures performed for HCC. Hospital volume during the survey period was determined using the unique identifier for each hospital and categorized into tertiles (low, intermediate and high volume), so that the numbers of patients in each group were approximately equal.

### Statistical analysis

Continuous variables are presented as median (i.q.r.), and categorical variables as numbers and frequencies. Univariable associations among groups were assessed using the χ^2^ test. Relationships between in‐hospital mortality and various factors, including patient characteristics and hospital volume, were assessed. Stepwise logistic regression analysis was used to model the concurrent effects of procedures and other factors on in‐hospital mortality. Statistical analyses were performed using SPSS^®^ software version 23.0 (IBM, Armonk, New York, USA). The threshold for significance was set at *P* < 0·050.

## Results

A total of 36 675 HCC patients underwent RFA at 811 hospitals during the study period. Clinical characteristics are summarized in *Table* 1. Some 66·4 per cent were men and the median age was 73 years; 62·7 per cent of all patients were aged years 70 or more, and 18·4 were aged at least 80 years.

**Table 1:** Patient characteristics

	No of patients (*n =* 36 675)*
Age (years)†	73 (66–78)
≤ 59	3399 (9·3)
60–69	10 268 (28·0)
70–79	16 256 (44·3)
≥ 80	6752 (18·4)
Sex ratio (F : M)	12 331 : 24 344
Charlson Co‐morbidity Index	
≤ 3	20 398 (55·6)
≥ 4	16 277 (44·4)
Aetiology	
Hepatitis B virus	3514 (9·6)
Hepatitis C virus	17 654 (48·1)
Hepatitis B + C virus	107 (0·3)
Alcohol	1400 (3·8)
Alcohol + hepatitis C virus	299 (0·8)
Alcohol + hepatitis B virus	39 (0·1)
Alcohol + hepatitis B + C virus	7 (0·0)
Others or data not provided	13 655 (37·2)

* Values in parentheses are percentages unless indicated otherwise;

† values are median (range).

**Figure 1:**
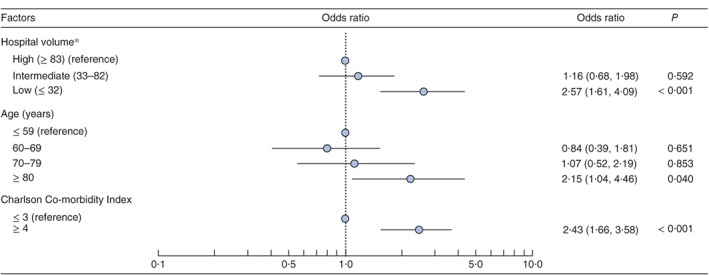
Multivariable logistic regression analysis of in‐hospital mortality following radiofrequency ablation (RFA). Odds ratios are shown with 95 per cent confidence intervals. *Defined according to the annual number of RFA procedures for hepatocellular carcinoma

*Table* 2 shows in‐hospital mortality rates following RFA. Hospitals with 83 or more, 33–82 and 32 or fewer RFA procedures were categorized as high‐, intermediate‐ and low‐volume hospitals respectively. The overall in‐hospital mortality rate following RFA was 0·31 per cent (115 of 36 675). In‐hospital mortality rates in high‐, intermediate‐ and low‐volume hospitals were 0·20 per cent (25 of 12 368), 0·23 per cent (29 of 12 405) and 0·51 per cent (61 of 11 902) respectively. This mortality rate was significantly higher in low‐volume hospitals (*P* < 0·001), older patients (*P* = 0·002) and those with higher CCI scores (*P* < 0·001). In multivariable analysis, in‐hospital mortality was significantly higher in low‐volume than in high‐volume hospitals (odds ratio 2·57, 95 per cent c.i. 1·61 to 4·09; *P* < 0·001). Higher in‐hospital mortality was also significantly associated with older age and higher CCI score (*Fig*. 1).

**Table 2:** Univariable analysis of factors influencing in‐hospital mortality after radiofrequency ablation

	Proportion who died	Mortality rate (%)	*P* †
Overall	115 of 36 675	0·31 (0·26, 0·38)	
Hospital volume*			< 0·001
High (≥ 83)	25 of 12 368	0·20 (0·13, 0·30)	
Intermediate (33–82)	29 of 12 405	0·23 (0·16, 0·34)	
Low (≤ 32)	61 of 11 902	0·51 (0·39, 0·66)	
Sex			0·95
F	39 of 12 331	0·32 (0·23, 0·43)	
M	76 of 24 344	0·31 (0·25, 0·39)	
Age (years)			0·002
≤ 59	9 of 3399	0·27 (0·12, 0·50)	
60–69	23 of 10 268	0·22 (0·14, 0·34)	
70–79	46 of 16 256	0·28 (0·21, 0·38)	
≥ 80	37 of 6752	0·55 (0·39, 0·76)	
Charlson Co‐morbidity Index			< 0·001
≤ 3	40 of 20 398	0·20 (0·14, 0·27)	
≥ 4	75 of 16 277	0·46 (0·36, 0·58)	

Values in parentheses are 95 per cent confidence intervals.

* Defined according to the annual number of radiofrequency ablation procedures for hepatocellular carcinoma.

† χ^2^ test.

## Discussion

RFA is a safe and effective treatment for HCC, with an in‐hospital mortality rate ranging from 0 to 1·8 per cent^3^ ^7^, ^19^, ^20^, ^21^, ^22^, ^23^, ^24^. Hospital volume is thought to influence various outcomes including in‐hospital mortality, even with minimally invasive treatments. A previous Japanese nationwide study of 11 688 patients^25^, however, failed to show a significant association between increasing hospital volume and decreasing mortality. It was felt that the sample size was too small to identify any association between hospital volume and RFA‐related mortality, as death occurred so rarely. Although a large number of patients was included in a meta‐analysis^12^, significant publication bias was identified^26^. The present study investigated the influence of hospital volume on in‐hospital mortality using a sample of 36 675 patients from a nationwide Japanese database.

The in‐hospital mortality rate was about 2·5‐fold higher in low‐volume hospitals than intermediate‐ and high‐volume hospitals. Volume–outcome relationships have been observed for many procedures and are often attributed to learning curve effects and standardization^15^ ^25^, ^27^, ^28^, ^29^. A previous study^30^ with more than 130 000 patients from the DPC database showed that better physician and nurse staffing were associated with lower rates of ‘failure to rescue’, independent of hospital volume. This implies that it may not be the single operator that makes the difference; rather all healthcare providers involved in the care of patients undergoing RFA may be implicated. In another DPC study^25^, increasing mortality following hepatectomy across high‐, intermediate‐ and low‐volume hospitals was observed, but the mortality difference between high‐ and intermediate‐volume hospitals was quite small. The present study had similar findings, suggesting a threshold for a minimum number of procedures.

A study from Taiwan^31^ also showed that patients who received first‐line RFA for HCC in a high‐volume hospital had better overall and cancer‐specific survival than those receiving RFA in low‐volume hospital. Taking this into account with the present findings, hospital volume may be associated with therapeutic performance as well as being safer in terms of procedure‐related mortality.

This study has several limitations. Although the database covers a large number of inpatient admissions, the study population may not be representative of all patients undergoing RFA in Japan because most participating hospitals were secondary and tertiary centres. Some important clinical data that may influence mortality, such as the size and location of tumours, were not available, although this might be offset by the likelihood that patients with more difficult problems tend to be referred to high‐volume hospitals. The database included only inpatient data, so mortality after leaving hospital could not be investigated. Many hospitals in Japan, however, provide both early postoperative care and subsequent rehabilitation during a single hospital stay. According to the Organization for Economic Co‐operation and Development's Health Statistics^32^, in 2014 the national average length of a hospital stay in Japan was 17·2 days, which is much longer than in other countries. It seems unlikely that there would be a difference between high‐ and low‐volume hospitals in terms of mortality after discharge. Finally, cause of death was not recorded in the database. Some in‐hospital deaths may not have been directly related to RFA, but all‐cause in‐hospital mortality is more robust in terms of objectivity than treatment‐related mortality assessed by operators. It is also seems unlikely that cause of death would differ according to hospital volume.

This study has confirmed that RFA for HCC is associated with an acceptably low mortality rate in Japan, but procedure‐related mortality is affected by patient background and hospital volume. Treatment of patients with complex problems and technically difficult lesions should be concentrated at high‐volume centres.
